# Evidence from the domestication of apple for the maintenance of autumn colours by coevolution

**DOI:** 10.1098/rspb.2009.0355

**Published:** 2009-04-15

**Authors:** Marco Archetti

**Affiliations:** Department of Zoology, University of OxfordSouth Parks Road, OX1 3PS Oxford, UK

**Keywords:** autumn colours, apple, aphid, photoprotection, coevolution, domestication

## Abstract

The adaptive value of autumn colours is still a puzzle for evolutionary biology. It has been suggested that autumn colours are a warning signal to insects that use the trees as a host. I show that aphids (*Dysaphis plantaginea*) avoid apple trees (*Malus pumila*) with red leaves in autumn and that their fitness in spring is lower on these trees, which suggests that red leaves are an honest signal of the quality of the tree as a host. Autumn colours are common in wild populations but not among cultivated apple varieties, which are no longer under natural selection against insects. I show that autumn colours remain only in the varieties that are very susceptible to the effects of a common insect-borne disease, fire blight, and therefore are more in need of avoiding insects. Moreover, varieties with red leaves have smaller fruits, which shows that they have been under less effective artificial selection. This suggests a possible trade off between fruit size, leaf colour and resistance to parasites. These results are consistent with the hypothesis that autumn colours are a warning signal to insects, but not with other hypotheses.

## 1. Introduction

### (a) Autumn colours

Many trees and shrubs of the temperate regions change their leaf colour in autumn. The biochemistry and the physiology of autumn colours are well known, but their adaptive value is still puzzling (Ougham *et al*. 2008; Archetti 2009*a*; Archetti *et al*. 2009). While yellow is due to the breakdown of chlorophyll, which unmasks the carotenoids already present in the leaf, red is due to anthocyanins, which are actively produced in autumn (Sanger 1971; Lee & Gould 2002; Archetti *et al*. 2009). What is the adaptive value of producing a pigment in leaves that are about to fall?

It is possible that anthocyanins protect the leaves against the damaging effects of light at low temperature, allowing the tree to reabsorb nutrients more efficiently, especially nitrogen (the photoprotection hypothesis: Feild *et al*. 2001; Hoch *et al*. 2001; Lee & Gould 2002). Evidence for this photoprotective effect exists but is controversial (Archetti *et al*. 2009). An alternative explanation is that autumn colours are the result of coevolution (the coevolution hypothesis: Archetti 2000; Hamilton & Brown 2001; Archetti & Brown 2004): red leaves might be a signal of quality for the insects that use the trees as a host. Many insect species, e.g. aphids, migrate to trees to lay their eggs in autumn during leaf colour change; the eggs, laid in twigs or crevices of the bark (not on the leaves), hatch in spring and the new generations of insects live on the tree before migrating to a different summer host. Because host quality can greatly affect insect fitness, there is strong selective pressure for the insects to find the most suitable host in autumn (Moran & Whitam 1990). According to the coevolution hypothesis, red leaves signal better chemical defences or worse nutritional capacity or any other characteristic that induces a lower fitness in the insects (Archetti 2000; Hamilton & Brown 2001; Archetti & Brown 2004). Insects, therefore, should prefer green trees in autumn and have higher fitness on these trees in spring.

Comparative analysis has shown that tree species with autumn colours are the ones with an evolutionary history of interaction with aphids (Hamilton & Brown 2001). Empirical studies have further shown that, within each tree species, aphids prefer individuals with green leaves and avoid individuals with red leaves in autumn (Furuta 1986; Archetti & Leather 2005; Rolshausen & Schaefer 2007; Ramirez *et al*. 2007) and that, although they probably lack a red photoreceptor, aphids can distinguish red from green by colour using the green–blue ratio (Döring *et al*. 2009). The fact that red is less attractive than green for aphids is no longer controversial (Archetti *et al*. 2009; Döring *et al*. 2009); evidence that insect fitness is lower on trees with red autumn leaves, however, is still lacking; this is the fundamental prediction of the coevolution hypothesis that remains to be tested.

Only two weak tests are available so far. In one case (Ramirez *et al*. 2007) no differential growth was found, but individual red leaves on completely green or yellow trees were used, rather than consistently red or green trees. In the second case, evidence is only indirect, based on the fact that aphids grow better on trees that drop their leaves later, which are known to have fewer autumn colours (Archetti & Leather 2005). There is also evidence (Karageorgou *et al*. 2008) that phenolics (common chemical defences against insects) are positively correlated with anthocyanins (red autumn colours), in a number of species.

Direct evidence that insect fitness is lower on trees with red autumn leaves, however, is lacking. It has been difficult to compare green and red trees because it is difficult to find a species with a clear polymorphism of autumn colours. Ideally, mutants for leaf colour should be used.

### (b) An ideal test of coevolution

Here, I test the coevolution hypothesis with domesticated and wild varieties of apple trees (*Malus pumila*). The cultivated apple, similar to some other fruit trees, originated from wild populations (sometimes called *M. sieversii*) in the mountains of Central Asia (CA), and populations of the same species (sometimes called *M. domestica*) evolved under domestication for many centuries (Harris *et al*. 2002). *Malus pumila* is infested by many species of insect pests, including aphids.

The domestication of apple offers exceptional opportunities to test the coevolution hypothesis. We can compare apple cultivars with green and red autumn leaves as we would do with individual mutants for colour. If red is a signal we would expect that aphids prefer trees with green leaves in autumn and that they have higher fitness in spring on trees with green autumn leaves. We can also compare domesticated and wild trees. Fruit trees under domestication have been artificially selected for productivity and taste, irrespective of their autumn colours, and they have not been under natural selection against insects. While cultivated trees are still attacked by parasites, their fitness and reproduction does not ultimately depend on their reaction to insects. If red is a signal we would expect it to be common in wild populations and not in cultivated varieties. We would also expect, as predicted by signalling theory, red varieties to be more vigorous or more in need of signalling.

These predictions are all necessary if the coevolution hypothesis is correct. By contrast, if red is simply the by-product of leaf senescence we would expect no difference in aphid behaviour and no difference in aphid fitness between trees with red and green leaves, no difference in colour between wild and cultivated varieties and no correlation between leaf colour and susceptibility to insect damage. If red is an adaptation for a more efficient recovery of nutrients we would expect that aphids prefer, and have higher fitness on, trees with red leaves in autumn, and no difference in colour between wild and cultivated varieties (Archetti 2007, 2009*a*; Archetti *et al*. 2009).

## 2. Material and methods

### (a) Apple varieties: leaf colour

Autumn leaf colour was recorded for all 2170 varieties (two individuals per variety) at the UK national fruit tree collection of the Brogdale Horticultural Trust, near Faversham, UK, in October and November 2007. Colour was also assessed for wild and cultivated trees in CA in October 2005: two wild populations, one (*n*=32) in the Zailiysky Alatau, south of Almaty, Kazakhstan; and one (*n*=42) in the Jalalabad Oblast, near Arslanbob in Kyrgyzstan, and an orchard near Tamga, Issyk-Kul, Kyrgyzstan, with trees (*n*=400) planted approximately 20 years ago. Colour was assessed by eye looking at the whole tree. Each cultivar was assigned a colour code: green (G); yellow (Y); or red (R) if the colour was present on more than 80 per cent of the leaves; GY, YR and GR if more than 80 per cent of the leaves belong to one of the two colours; GYR for uncertain cases. Rougher estimates (per cent of green, yellow and red) come from the following locations and years: the US national fruit tree collection of the US Department of Agriculture's Agricultural Research Service (USDA—ARS) in Geneva, NY (approx. 200 domesticated trees and approx. 60 trees from CA, October 2007; from photographs provided by P. Forsline); orchards in Kent, UK (November 2006–2007); Trentino, Italy (November 2007); Umbria, Italy (November 2005–2007); Talca, Chile (April 2008); Samarkand, Uzbekistan (October 2005).

### (b) Apple varieties: other data

Data for apple varieties were retrieved from the Germplasm Resources Information Network database of the USDA—ARS (http://www.ars-grin.gov/npgs/acc/acc_queries.html), which provides phenological, morphological and biochemical information about all the apple varieties present in the US collection. The varieties for which the colour category was available and the varieties from CA found in the US collection were used in the analysis of the following descriptors: FBSHNAT (580 varieties), natural occurrence of fire blight (score: 1–5; from very resistant to very susceptible); FRTWEIGHT (999 varieties), fruit weight (score: 1–9; from less than 50 g to more than 400 g); TREEVIGOR (404 varieties), tree vigour when grown on a vigorous rootstock (score: 1–4; from small to very vigorous); FRTFLSHFLA (2123 varieties), fruit flesh flavour (aromatic, sweet, subacid, acid and astringent); FRTOVERCOL (1927 varieties), fruit over colour.

### (c) Aphid preference

I used the aphid *Dysaphis plantaginea*, a common host-alternating aphid for which *M. pumila* is a winter host. Abundance of aphids (winged individuals) in autumn was assessed at Brogdale on 5 different days, once a week, during October 2007, on 120 individual trees with different leaf colours (40 green, 40 yellow and 40 red), belonging to different varieties but with the same age and size, and at the same distance from one another. I used varieties whose colour could be classified unequivocally as red, green or yellow (Döring *et al*. 2009). Aphid species found on the trees were 78 per cent *D. plantaginea*, 14 per cent *Aphis pomi*, 8 per cent uncertain; only *D. plantaginea* was used for the analysis.

### (d) Aphid fitness

Performance of aphids (*D. plantaginea*) in spring was assessed at the nearby Jeskyns Community Woodland, near Cobham, UK, where trees originating from the Brogdale collection had been planted 3 years before the test. All the trees used for the experiments were the same age and grow in the same soil and conditions. No insecticide had been used in the previous 3 years, and the site had been left unmanaged. All leaves were green in spring. To measure aphid fitness, a colony of *D. plantaginea* was chosen in spring on an unsprayed apple tree in an organic orchard at the nearby East Malling Research Station. Virginoparae of the same age from this colony were placed on different trees (one aphid per tree) at Jeskyns on the same day at the beginning of May 2008, and monitored until the end of June 2008 (virginoparae were transferred from the branch of the original tree directly onto a leaf of the new tree and placed inside a clip cage using a fine brush, all within one hour). An individual was considered successful if it managed to complete the cycle leading to the production of an adult winged summer migrant. The total number of aphids tested was 260 (96 on red, 144 on green, and 20 on yellow trees). Only trees without natural colonies (easily spotted by the presence of curled leaves) were used in experiments with clip cages.

## 3. Results and discussion

### (a) Aphids avoid trees with red leaves in autumn

**Figure 1:**
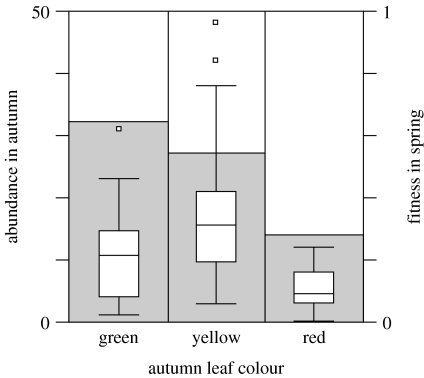
Aphids avoid trees with red leaves in autumn and have higher fitness in spring on trees with green autumn leaves. Box and whisker plots showing the median, quartiles and outliers of aphid abundance per tree (left axis) on trees with different leaf colours in autumn. Trees with red leaves (*n*=40) attracted on average less aphids than trees with green leaves (*n*=40; Mann–Whitney *U*-test: *U*=1212, *p*<0.0001) or yellow leaves (*n*=40; Mann–Whitney *U*-test: *U*=1445, *p*<0.0001) in autumn. Yellow attracted more aphids than green (Mann–Whitney *U*-test: *U*=1103, *p*<0.01). The grey areas (right axis) show the fitness of aphids in spring, measured as the proportion of virginoparae that managed to produce a summer migrant. Aphid fitness on trees with red autumn leaves was lower than on trees with green autumn leaves (*n*=237, *Χ*^2^=25.3, *p*<0.0001) and on trees with yellow autumn leaves (*n*=116, *Χ*^2^=4.9, *p*<0.05); green and yellow do not differ (*n*=161, *Χ*^2^=0.4, *p*=0.52).

Consistent with the coevolution hypothesis, I found that the apple varieties with red leaves attracted fewer winged aphids in autumn than the varieties with green or yellow leaves (figure 1). It is now known that, although they probably lack a high wavelength (‘red’) photoreceptor (Döring & Chittka 2007), aphids can clearly differentiate red from green and yellow, using the blue–green ratio (Döring *et al*. 2009). The fact that aphids prefer green over red leaves, however, does not necessarily mean that red is a signal. Red leaves might attract fewer insects simply because they exploit an inherent bias in insect colour vision, that is red might be a form of camouflage that makes leaves more cryptic to insects (Döring *et al*. 2009). For red to be a signal it must have coevolved with colour preference. This can be tested indirectly by measuring the fitness of aphids in spring.

### (b) Aphids have higher fitness in spring on trees with green and yellow autumn leaves

If red is an honest signal of the tree's quality as a host for insects, we would expect aphids to have higher fitness on trees that have green autumn leaves. If autumn colours were non-adaptive, or an adaptation to reabsorb more nitrogen, there would be no reason why aphids, which in fact need nitrogen, prefer trees with green leaves and perform better on these trees in spring. Here, I tested this prediction directly (figure 1) and, as expected by the coevolution hypothesis, the proportion of aphids that managed to grow and develop was significantly lower on trees with red autumn leaves (29% survival) than on trees with green autumn leaves (61% survival) or yellow autumn leaves (55% survival).

### (c) Autumn colours are less common under domestication

The ideal test of the coevolution hypothesis would be to let populations of the same species evolve with and without insect pests for many generations: if autumn colours are a signal to insects, we would expect red coloration to be lost in the populations evolving without insects. This experiment would take too long to perform, but a similar test was actually initiated *ca* 2000 years ago with the domestication of fruit trees.

Most species in the genus *Malus* (apple) have red leaves in autumn (Archetti 2009*b*), which suggests that red was the ancestral autumn colour for *M. pumila*. If red is a signal to insects, we would expect autumn colours to be common in wild populations of *M. pumila* but not under domestication, because cultivated apple trees are no longer under selection against parasites. If autumn colours were simply a by-product of leaf senescence there would be no reason why they should be lost under domestication; if, on the other hand, red evolved because it allows a more efficient resorption of nutrients, autumn colours should also be maintained under domestication.

**Figure 2:**
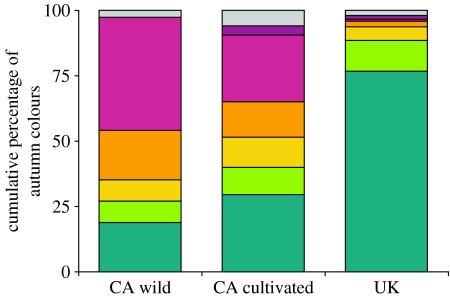
Autumn colours are less common under domestication. Red autumn colours (R, YR and GR, opposed to G, GY and Y) are more common among wild (*n*=74) than among cultivated (*n*=400) trees in CA (*Χ*^2^=10.44, *p*<0.005), and more common in CA (cultivated and wild) than in the UK: (*n*=2170; *p*≪0.0001). G: green; Y: yellow; and R: red.

Consistent with the coevolution hypothesis, I found that, while 62.2 per cent of the trees in wild populations of *M. pumila* in CA (Kyrgyzstan and Kazakhstan) change their leaf colour to red in autumn, only 2.8 per cent of the 2170 English cultivated varieties turn red. Loss of red autumn colours is also evident in cultivated varieties in Central Asia, where 39.0 per cent of the trees in orchards change their colour to red, which is significantly lower than in the wild, although not so evident as in the UK varieties (figure 2). An estimation of autumn colours in other locations and years reveals a similar pattern. In the US collection in 2007, less than 5 per cent of the US varieties turned red and 15 per cent yellow, whereas 30 per cent of the Central Asian wild varieties growing in the US collection turned red and 40 per cent yellow. In other orchards in Italy, Chile and the UK 20 per cent turned yellow between 2005 and 2008 and no red trees were found; in an orchard in Uzbekistan 40 per cent were red and 40 per cent yellow in 2005. This suggests that autumn colours are not simply a reaction to different environmental or geographical conditions but have been lost under domestication.

### (d) Varieties with red autumn leaves have higher susceptibility to fire blight

Signalling theory predicts that the individuals that signal, or signal more than the average, are either more vigorous (need being equal, because they can afford more easily to pay the cost of the signal) or more in need of signalling (vigour being equal, because they have a greater advantage from signalling; Maynard-Smith & Harper 2003). I tested this prediction using available data for vigour (descriptor: TREEVIGOR) of the apple varieties in the US national fruit tree collection. Red varieties (average: 3.40, *n*=25) do not have a significantly higher average vigour than green (average: 3.33, *n*=281; Mann–Whitney *U*-test: *U*=4279, *p*=0.44) or yellow (average: 3.27, *n*=71; Mann–Whitney *U*-test: *U*=1056, *p*=0.62) varieties. If autumn colours are a signal, therefore, we would expect that red varieties are more in need of signalling.

The need to avoid insects is more difficult to quantify than tree vigour, but it can be estimated by measuring the amount of damage induced by the insects. Varieties that are affected more by the deleterious effects of insect attack are the ones that need to avoid insects more. Insect-induced damage may depend on the amount of infestation, especially for leaf chewers, but for phloem sucking insects like aphids it is more likely to be related to the transmission of bacterial, fungal or viral diseases. Therefore a good indicator of the need to avoid insects for a tree is its susceptibility to an insect-borne disease.

**Figure 3:**
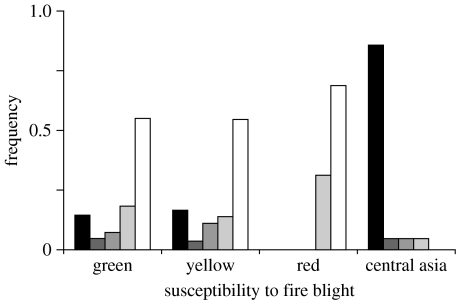
Varieties with red autumn leaves have higher susceptibility to fire blight. The varieties from CA (*n*=42; average susceptibility, 1.29) have lower (Mann–Whitney *U*-test, *p*≪0.0001) susceptibility to fire blight than the US varieties (*n*=538; average susceptibility, 3.97). Among US varieties, the red ones (*n*=32; average susceptibility, 4.69) are more susceptible to fire blight than the green ones (*n*=398; average susceptibility, 3.94; Mann–Whitney *U*-test: *U*=7772, *p*<0.05). Susceptibility score: 1–5; from very resistant to very susceptible. Black, 1; dark grey, 2; grey, 3; light grey, 4; and white, 5.

I use susceptibility to fire blight (descriptor: FBSHNAT) as an indicator of signalling need. Fire blight is a common and important contagious disease of apple trees induced by *Erwinia amylovora*, a bacterium transmitted by aphids and other insects. Varieties more susceptible to fire blight are the ones more in need of avoiding insects, and therefore more in need of signalling. An analysis of different varieties in the US national fruit tree collection shows that the varieties from CA have an extraordinarily lower susceptibility to fire blight than the US varieties, irrespective of colour (figure 3). This is not surprising: since fire blight originated in USA, it may have not adapted to attack the Central Asian varieties.

What is interesting is that among the US cultivated varieties, the red ones turn out to be on average significantly more susceptible to fire blight than the green ones (figure 3). Only very susceptible varieties have red autumn colours. This suggests that the cultivars that maintain autumn colours are the ones more in need of avoiding insects, as predicted by the coevolution hypothesis. This observation, again, cannot be explained if red is non-adaptive or if it is an adaptation to reabsorb more nitrogen.

### (e) Varieties with red autumn leaves have lower fruit size and more astringent taste

**Figure 4:**
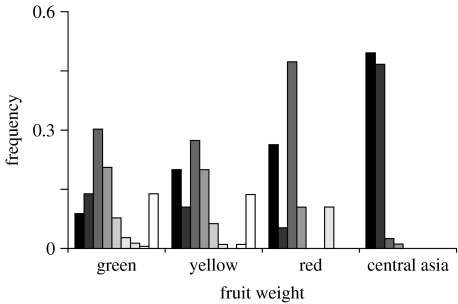
Varieties with red autumn leaves have lower fruit size. The varieties from CA (*n*=512, average weight: 1.55) have lower fruit weight than the US varieties (*n*=487, average weight: 3.94; Mann–Whitney *U*-test: *U*=216 529, *p*<0.0001). Red cultivars in the US (*n*=19, average weight: 2.95) have significantly lower fruit size than green cultivars (*n*=360, average weight: 4.05; Mann–Whitney *U*-test: *U*=4407, *p*<0.05). Fruit weight score: 1–9 from less than 50 g (black) to more than 400 g (white).

The domesticated apple evolved under artificial selection for improved fruit size and taste. It is not surprising, therefore, that the varieties from CA have lower fruit size (descriptor: FRTWEIGHT) than the domesticated varieties, irrespective of colour (figure 4). It is interesting, however, to compare cultivated varieties with different autumn colours. Because increasing fruit size has been the main selective pressure under domestication, varieties that have undergone less efficient selection are expected to have smaller fruits. If red varieties have evolved more slowly under domestication than green varieties, therefore, we would expect that they have smaller fruits on average. Consistent with this prediction, I found that red cultivars have significantly lower fruit size than green cultivars (figure 4). This suggests that there is a trade-off in natural conditions between fruit size, autumn colours and reaction against insects.

It is also interesting that fruits with astringent flavour are more common (compared to all other flavours–descriptor: FRTFLSHFLA) in varieties with red leaves than in varieties with green (*n*=307, *Χ*^2^=19.5, *p*<0.0001) or yellow leaves (*n*=106, *Χ*^2^=6.5, *p*<0.01), whereas green and yellow varieties do not differ (*n*=371, *Χ*^2^=1.75, *p*=0.19); the varieties from CA have more astringent fruits than the cultivated green (*n*=1985, *Χ*^2^=122, *p*<0.0001) or yellow varieties (*n*=1784, *Χ*^2^=30.5, *p*<0.0001) but not more than the red varieties (*n*=1720, *Χ*^2^=0.75, *p*=0.39). Since apple varieties have been selected against astringent flavour, this also supports the idea that cultivars with red leaves are more similar on average to their wild ancestors.

Fruit colour (descriptor: FRTOVERCOL) however does not have any significant correlation with autumn leaf colours; indeed, most cultivars have red or orange fruits.

## 4. Conclusion

The five results I have shown are consistent with the main predictions of the coevolution hypothesis:

- Aphids are more abundant on green and yellow autumn leaves than on red leaves.
- Aphids have higher fitness in spring on trees with green and yellow autumn leaves than on trees with red leaves.
- Autumn colours are common in wild varieties but rare under domestication.
- Only varieties with high susceptibility to fire blight have red autumn leaves.
- Varieties with red autumn leaves have smaller fruits and more astringent taste.

These results are not expected, nor even in direct contradiction with the photoprotection hypothesis, nor with the idea that autumn colours are simply a by-product of leaf senescence. The photoprotection hypothesis has been for a long time the only candidate for an adaptive explanation of autumn colours, but a survey of the literature reveals that only half of the available empirical evidence supports it while the other half does not (Archetti 2009*a*; Archetti *et al*. 2009). A reassessment of this contrasting evidence is necessary before we accept or reject photoprotection as a possible explanation for autumn colours (Ougham *et al*. 2008).

The coevolution hypothesis, on the other hand, has been proposed more recently (Archetti 2000; Hamilton & Brown 2001) and has been subject to extensive criticism, but the tests performed so far (Archetti *et al*. 2009; Archetti 2009*a*) and the results presented here are all consistent with its predictions. Clearly, the coevolution hypothesis is not restricted to aphids, and colour preference in autumn should be tested in more insect–plant interactions.

I have shown that autumn colours have been lost during domestication in apple trees. Apricot (*Prunus armeniaca*) and walnut (*Juglans regia*) trees also turn red–orange in wild populations in CA, but are green or yellow in cultivations in Europe. The idea that autumn colours are lost under domestication should be tested with these and other species, as well as the suggestion that this is due to the fact that signalling is no longer necessary in cultivated varieties.

Colour as a signal is very common in nature, both in mutualistic interactions, e.g. in flowers to attract pollinators; and in antagonistic interactions, e.g. to warn off predators. Autumn colours seem to be another example.
